# Pre-ECMO characteristics, organ dysfunction and exploratory mortality prediction in COVID-19 versus non-COVID-19 ARDS treated with veno-venous ECMO: a retrospective cohort study

**DOI:** 10.1186/s12871-026-04306-9

**Published:** 2026-10-07

**Authors:** Mirja Mittermaier, Jan Adriaan Graw, Alexander H. Thieme, Johann Lieberwirth, Oliver Hunsicker, Daniel Zickler, Jan M. Kruse, Alexander Uhrig, Panagiotis Pergantis, Carmen Garcia, Felix Machleidt, Till Jacobi, Victoria Bünger, Michaela Ludmilla Merten, Micha Landoll, Christian Karagiannidis, Leif-Erik Sander, Kai-Uwe Eckardt, Claudia Spies, Martin Witzenrath, Steffen Weber-Carstens, Mario Menk, Holger Müller-Redetzky

**Affiliations:** 1 Department of Infectious Diseases, Respiratory Medicine and Critical Care Medicine, Charité – Universitätsmedizin Berlin, Corporate Member of Freie Universität Berlin and Humboldt-Universität zu Berlin, Charitéplatz 1, 10117 Berlin, Germany; 2 Berlin Institute of Health at Charité – Universitätsmedizin Berlin, Charitéplatz 1, 10117 Berlin, Germany; 3 Department of Anaesthesiology, Friedrich-Alexander-Universität Erlangen-Nürnberg (FAU), Universitätsklinikum Erlangen, 91054 Erlangen, Germany; 4 Center for Biomedical Informatics Research (BMIR), Department of Biomedical Data Science, Stanford University, Stanford, CA USA; 5 Department of Medicine, Stanford University, Stanford, CA USA; 6 Department of Radiation Oncology, Charité – Universitätsmedizin Berlin, Corporate Member of Freie Universität Berlin and Humboldt-Universität zu Berlin, Augustenburger Platz 1, 13353 Berlin, Germany; 7 Department of Anesthesiology and Intensive Care Medicine, Charité – Universitätsmedizin Berlin, Corporate Member of Freie Universität Berlin and Humboldt-Universität zu Berlin, CCM/CVK, Augustenburger Platz 1, 13353 Berlin, Germany; 8 Department of Nephrology and Medical Intensive Care, Charité – Universitätsmedizin Berlin, Corporate Member of Freie Universität Berlin and Humboldt-Universität zu Berlin, Charité-Universitätsmedizin, Augustenburger Platz 1, 13353 Berlin, Germany; 9 ARDS/ECMO Centrum Charité, Charité – Universitätsmedizin Berlin, Corporate Member of Freie Universität Berlin and Humboldt-Universität zu Berlin, Charitéplatz 1, 10117 Berlin, Germany; 10 Department of Pneumology and Critical Care Medicine, ARDS and ECMO centre, Cologne- Merheim Hospital, Kliniken der Stadt Köln gGmbH, Cologne, Germany; 11 Witten/Herdecke University, Witten, Germany; 12 Department of Cardiovascular Engineering, Institute of Applied Medical Engineering, Medical Faculty, RWTH Aachen University, Aachen, Germany; 13 Institute for Computational Biomedicine, Medical Faculty, RWTH Aachen University, Aachen, Germany; 14 German Center for Lung Research (DZL), Berlin, Germany; 15 Department of Anesthesiology and Intensive Care Medicine, Faculty of Medicine, University Hospital Carl Gustav Carus, TU Dresden, Dresden, Germany

**Keywords:** Extracorporeal membrane oxygenation (ECMO), COVID-19, RESP score, Acute respiratory distress syndrome, SARS-CoV-2, CEMP score

## Abstract

**Objective:**

The transferability of pre-ECMO characteristics and established mortality prediction tools across COVID-19 (C-19) and non-COVID-19 (nC-19) acute respiratory distress syndrome (ARDS) remains uncertain. We compared clinical characteristics, organ dysfunction, respiratory support and outcome in a large cohort of patients treated with veno-venous extracorporeal membrane oxygenation (vvECMO), and explored mortality prediction in C-19 ARDS.

**Methods:**

This retrospective single-center cohort study included *n* = 600 vvECMO patients (C-19: *n* = 245, nC-19: *n* = 355) hospitalized at the Charité ARDS ECMO Center from 01/2007 to 12/2018 (nC-19) and 03/2020–01/2022 (C-19). Pre-ECMO clinical and laboratory variables were compared between cohorts and by ICU survival. RESP score performance was assessed, and an exploratory C-19 ECMO mortality prediction (CEMP) score was derived from pre-ECMO variables using an explainable machine-learning workflow with SHapley Additive exPlanations (SHAP).

**Results:**

C-19 patients were older (median 57.0 vs. 51.0 years, *p* < 0.0001), more often male (74.7% vs. 66.8%, *p* = 0.0337), and had a higher BMI (29.3 vs. 26.0 kg/m², *p* < 0.0001). nC-19 patients had a higher CCI (3 vs. 2, *p* = 0.0137) and SOFA score (12 vs. 11, *p* = 0.0004). Septic shock before vvECMO initiation was more prevalent in nC-19 patients (65.4% vs. 13.9%, *p* < 0.0001), and nC-19 patients had higher median baseline bilirubin, creatinine, and lactate levels (all *p* < 0.0001). nC-19 patients had higher median PEEP, pPeak, and plateau pressure than C-19 patients, whereas their PaO₂/FiO₂ ratio was lower (*p* < 0.0001). Duration of vvECMO support was longer in C-19 patients (26.0 vs. 12.6 days, *p* < 0.0001); ICU mortality did not differ significantly (55.1% vs. 47.9%, *p* = 0.0966). RESP showed limited discrimination in C-19 patients, and the CEMP derivation analysis yielded an apparent AUC of 0.757 (95% CI 0.69–0.82).

**Conclusion:**

This large single-center comparative vvECMO cohort highlights differences between C-19 and nC-19 ARDS in organ dysfunction burden, respiratory characteristics, and treatment duration. ICU mortality did not differ significantly between groups. RESP showed limited discrimination in C-19; CEMP remains exploratory and requires calibration and independent external validation before clinical application.

**Supplementary Information:**

The online version contains supplementary material available at https://doi.org/10.1186/s12871-026-04306-9.

## Introduction

The COVID-19 (C-19) pandemic led to an unprecedented increase in the use of veno-venous extracorporeal membrane oxygenation (vvECMO) for C-19-associated acute respiratory distress syndrome (ARDS). The length of ECMO treatment and of mechanical ventilation (MV) has been shown to be prolonged in C-19 patients compared to “typical” ARDS patients [1], suggesting a C-19-related phenotype.

Prior work from our group described fibroproliferative changes in late-stage severe C-19 ARDS [2], possibly contributing to prolonged treatment. However, previous studies have identified various factors, such as the hyperinflammatory pre-ECMO phenotype, associated with higher mortality in both C-19 and non-COVID-19 (nC-19) ARDS patients; phenotypic differences between and within the two cohorts are not yet fully understood [3–5]. The Respiratory ECMO Survival Prediction (RESP) score [6] predicts survival well in mixed-etiology ARDS treated with vvECMO, but its transferability to C-19 ARDS remains uncertain.

Direct comparative ECMO studies have yielded heterogeneous mortality estimates, emphasizing the relevance of comparator etiology, case mix, patient selection, and treatment era [7–9].

This study aimed to characterize differences in pre-ECMO characteristics, organ dysfunction and outcomes between C-19 ARDS and nC-19 ARDS treated with vvECMO. We further compared survivors and non-survivors within both cohorts and derived an exploratory C-19 vvECMO mortality prediction (CEMP) score for ICU mortality using an explainable machine-learning approach.

## Methods

### Study design and study population

**Fig. 1:**
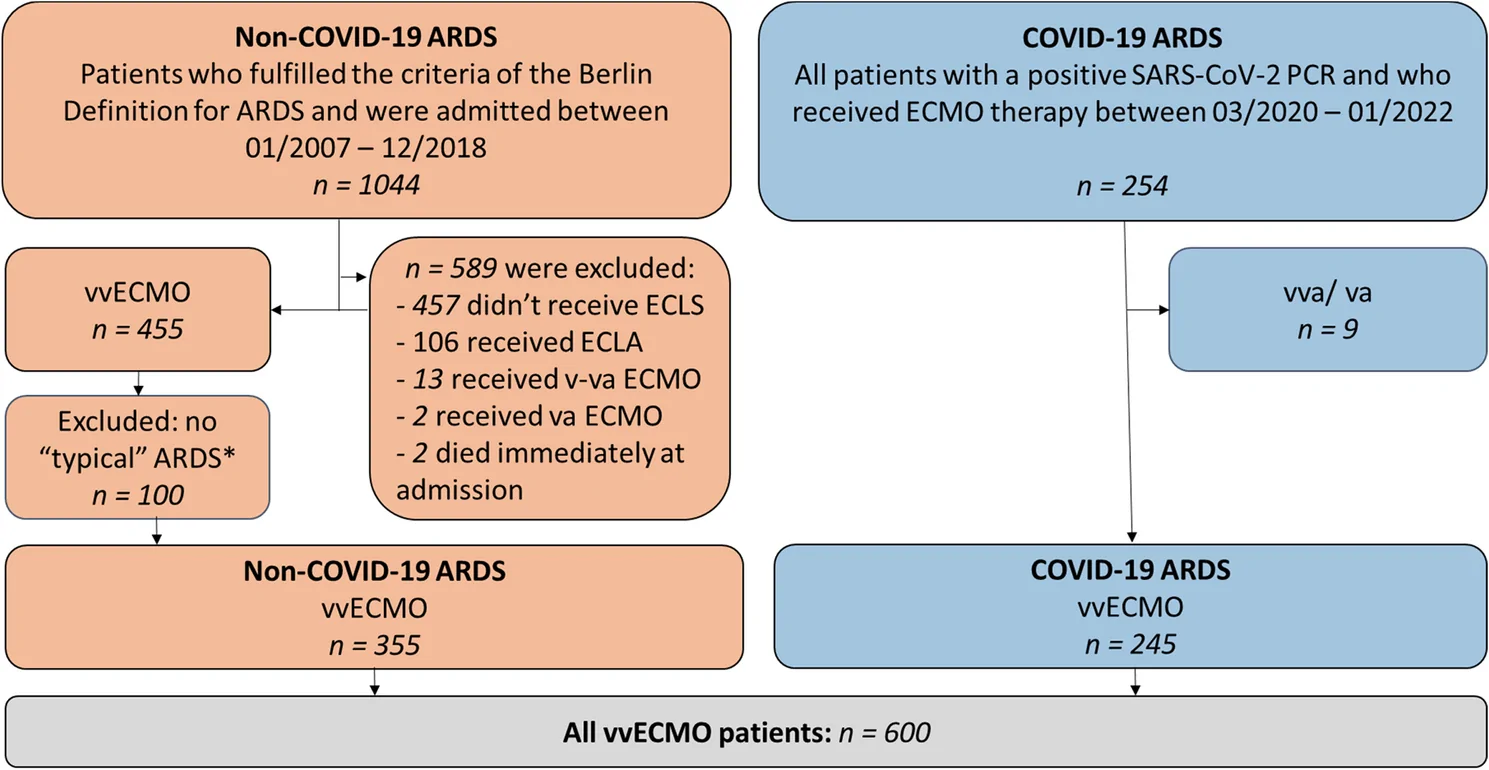
Flowchart of patient recruitment: The study population comprises *n* = 600 vvECMO ARDS patients, and includes a non-COVID-19 and a COVID-19 cohort. Non-COVID-19 cohort: Between 01/2007 and 12/2018, *n* = 1044 ECMO patients were treated in the Charité ARDS ECMO Centrum. Only patients requiring solely vvECMO therapy were included in this study, and patients with ECLA or vaECMO were excluded. *Further, patients with one of the reasons for respiratory failure were excluded from the study: secondary sepsis, trauma, pancreatitis, bleomycin-induced pneumonitis, and cystic fibrosis. COVID-19 cohort: Between 03/2020 and 01/2022, *n* = 245 COVID-19 patients requiring vvECMO support due to respiratory failure were treated in the Charité ARDS ECMO Centrum. Abbreviations: ARDS: Acute Respiratory Distress Syndrome; vvECMO: veno-venous extracorporeal membrane oxygenation; ECLA: Extracorporeal lung assist; ECLS: Extracorporeal life support; vaECMO: veno-arterial ECMO; vvaECMO: veno-venous-arterial ECMO

A single-center retrospective analysis of *n* = 600 adult (aged ≥ 18 years) patients requiring vvECMO due to ARDS, consisting of *n* = 245 C-19 and *n* = 355 nC-19 patients, was performed. Patients enrolled in the C-19 cohort (*n* = 245) treated between March 1st, 2020, and January 31st, 2022, and requiring vvECMO all had a positive polymerase chain reaction (PCR) for SARS-CoV-2 infection. Only patients with a completed ICU outcome (discharge or death) were included. The nC-19 cohort comprised patients admitted for vvECMO therapy between January 1st, 2007, and December 31st, 2018. The two cohorts were therefore non-contemporaneous. All patients were treated in ICUs of the ARDS ECMO Center of the Charité-Universitätsmedizin Berlin, a tertiary referral center for ARDS and ECMO therapy (Supplementary Methods provide detailed information on vvECMO indication and external vvECMO initiation). Patients requiring vaECMO and vvaECMO therapy were excluded from this study, as well as patients with one of the following reasons for respiratory failure: secondary sepsis, trauma, pancreatitis, bleomycin-induced pneumonitis, and cystic fibrosis (Fig. 1). The nC-19 cohort was etiologically heterogeneous: bacterial *n* = 120 (33.8%), non-SARS-CoV-2 viral *n* = 70 (19.7%), aspiration *n* = 45 (12.7%), fungal *n* = 44 (12.4%), mixed *n* = 30 (8.5%), and unknown *n* = 46 (12.9%) (Table 1; Supplementary Table 1), broadly reflecting the etiologic spectrum reported in other ARDS cohorts [10].

Pre-ECMO clinical variables were collected 2–8 h before ECMO initiation, and laboratory parameters (creatinine and bilirubin) were collected on the same day as ECMO initiation (Table 1). Missing data (e.g., blood gas analysis data of patients in whom vvECMO therapy was initiated at an external hospital by one of our mobile teams) are presented in Supplementary Tables 2 and 3. Of note, total hours of dialysis and nitric oxide (NO) inhalation were solely obtained during patients’ stay in ICUs of Charité. If patients received either therapy at an external hospital before referral, duration of therapy was not included in this analysis. Neuromuscular blockade data were unavailable for the nC-19 cohort, precluding a between-cohort comparison. Although lung-protective ventilation strategies represented standard clinical practice during both treatment periods, the available dataset did not permit consistent patient-level assessment of tidal volume indexed to predicted body weight, driving pressure, respiratory-system compliance, or adherence to a standardized lung-protective ventilation protocol. Consequently, adherence to lung-protective ventilation could not be formally verified from the available data. Missingness reflected measurement and documentation practices as well as external ECMO initiation. Analyses were based on available cases, and no multiple imputation or formal missing-data sensitivity analysis was performed.

**Table 1:**
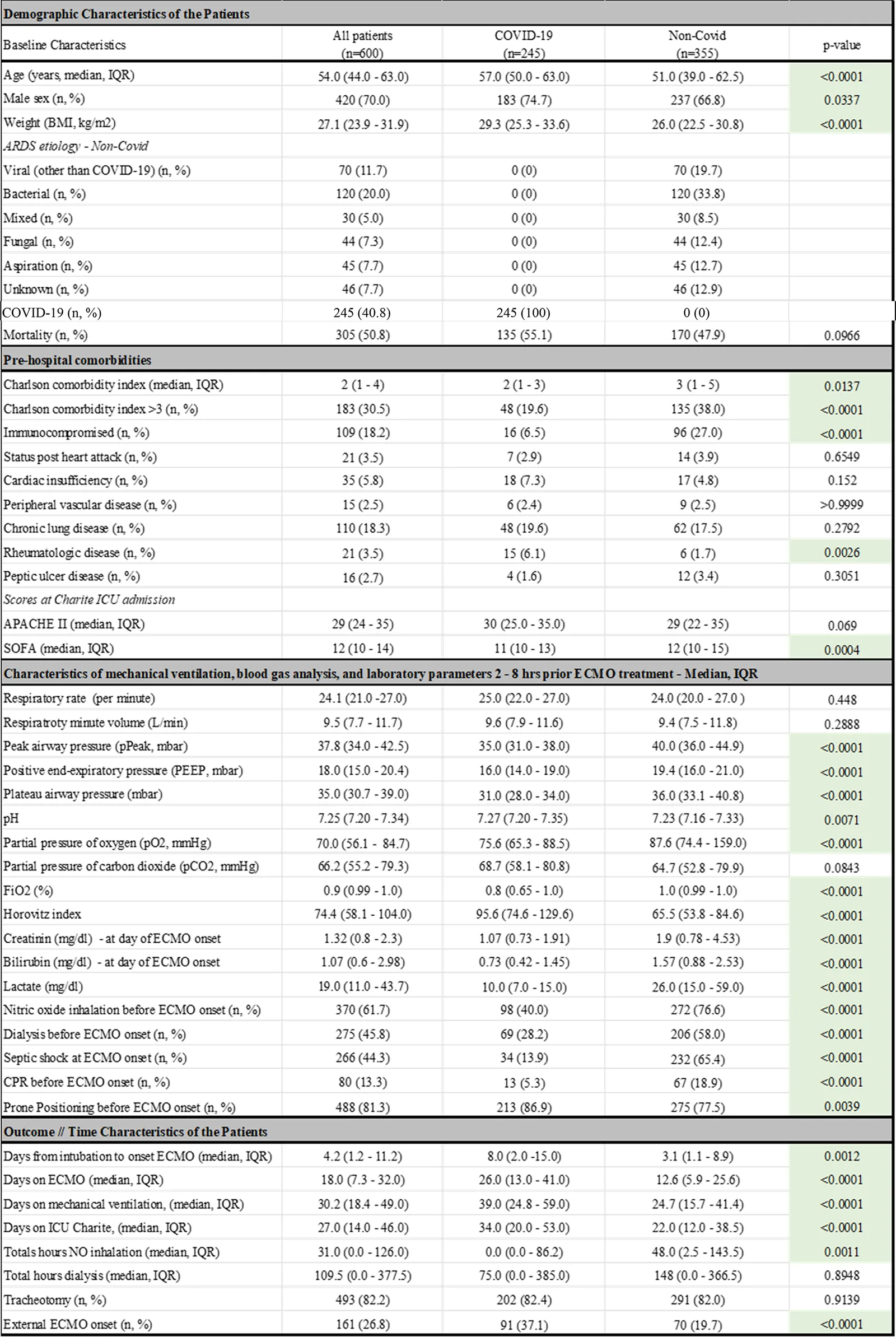
Patient characteristics

### RESP score calculation

**Fig. 2:**
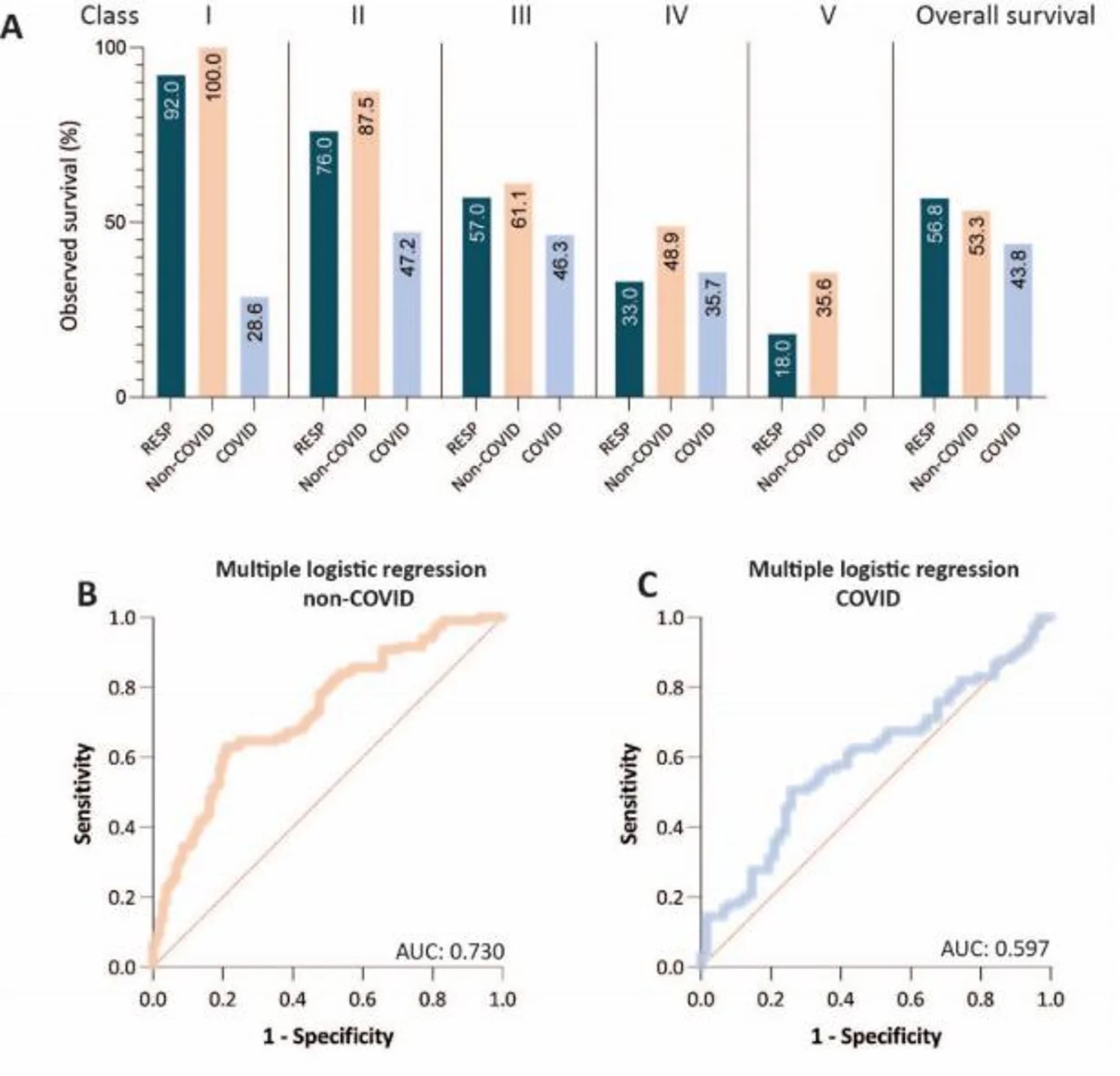
RESP score in COVID-19 and non-COVID-19 vvECMO patients. **A**. Individual RESP scores were calculated in the COVID-19 and non-COVID-19 cohorts. For score calculation, patients with all remaining RESP inputs available after zero coding of systematically unavailable items were used (COVID-19: n=147, non-COVID-19: n = 229). Observed survival (%) per RESP risk class of the original RESP score (green), the COVID-19 cohort (blue), and the non-COVID-19 cohort (red) are displayed. Further, like in the original RESP score paper, multivariable analysis was performed to identify which parameters are associated with survival (please see results in the main text) and to assess the area under the ROC curve (AUC) of the RESP score in the non-COVID-19 (**B**) and COVID-19 cohort (C). Zero coding is not equivalent to documented absence and may bias performance estimates

Individual patient Respiratory ECMO Survival Prediction (RESP) scores were calculated in accordance with Schmidt et al. [6] (Supplementary Table 4). Following the RESP score calculation, patients were categorized into the appropriate survival risk class (risk class I to V, Supplementary Table 5A-B). Observed survival by RESP risk class is displayed in Fig. 2 for patients with all remaining score inputs available after predefined zero coding. For C-19 patients, all cases were scored as having viral pneumonia. In the nC-19 cohort, neuromuscular blockade and bicarbonate data were unavailable before ECMO initiation. In the C-19 cohort, data for bicarbonate therapy were not collected. Based on the example of the original RESP score publication, a score of zero was assigned to these missing items. Assigning zero points to unavailable items is not equivalent to documented absence and may bias score distributions and discrimination. The primary RESP analyses therefore included patients with all remaining score inputs available (C-19, *n* = 147; nC-19, *n* = 229).

### Machine learning and C-19 ECMO mortality score development

**Fig. 3:**
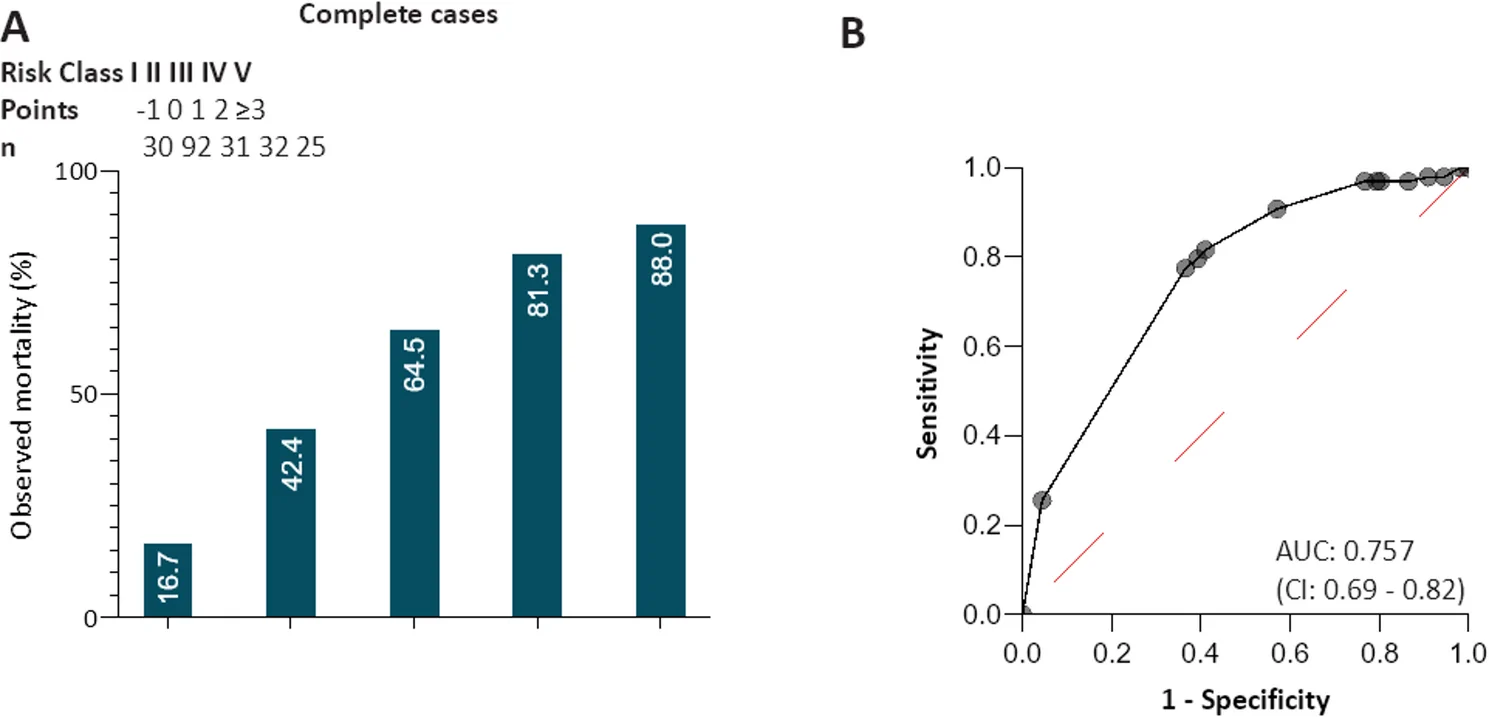
COVID-19 ECMO Mortality Prediction (CEMP) score performance: **A**: The bar graph displays the observed ICU mortality in percent per score risk class of the 210 complete cases. Further, information about points necessary to get assigned to a risk class and the number of patients is given. **B**: Apparent discrimination of the CEMP score in the 210 complete cases yielded an area under the ROC curve of 0.757 (95% CI 0.69–0.82); no optimism correction or calibration was performed

Because the RESP score showed limited discrimination in COVID-19 patients, a machine-learning workflow was applied. A classifier was built, feature contributions were examined, and the CEMP score was calculated (Supplementary Methods). Ventilation and blood-gas variables had missing values (Supplementary Tables 2 and 3). Individual CEMP scores were calculated in complete cases, and apparent performance was assessed (Fig. 3; Table 2). The primary CEMP analysis was restricted to 210 complete cases.

**Table 2:**
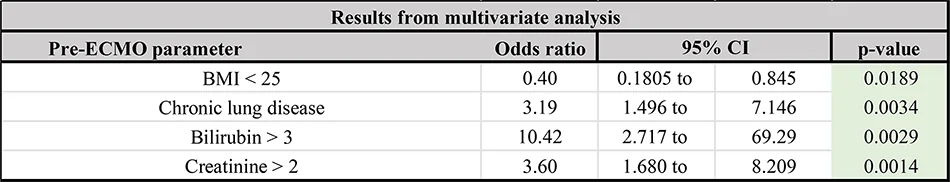
Exploratory multivariable associations of the CEMP components with ICU mortality: Multivariable analysis was performed in complete cases with the pre-ECMO parameters comprising the CEMP score, and associations with mortality were estimated for each component in the same complete-case cohort; this is not a validation analysis

Exploratory CEMP score assessment: Multivariable analysis with the four CEMP components estimated their associations with mortality in the same complete-case cohort (Table 1; Supplementary Methods).

Model performance assessment: Candidate variables were screened for associations with survival in the overall C-19 cohort before the 80:20 training/test split; therefore, the reported test-set performance does not represent fully independent internal validation and may be optimistic. The XGBoost test-set accuracy is reported with an exact binomial 95% confidence interval. The simplified CEMP was constructed as a point-based risk-stratification score and did not provide prespecified individual absolute mortality probabilities; calibration slope, calibration intercept, and Brier score were therefore not part of the original score assessment.

### Statistics

Descriptive statistics are expressed as median (IQR) for continuous variables and as percentages for categorical variables; missing data are summarized in Supplementary Tables 2 and 3. Continuous variables were compared with a two-tailed Student’s t-test or the Mann-Whitney U test, as appropriate. Normal Gaussian distribution was verified using the D’Agostino-Pearson normality test, Anderson-Darling test, Shapiro-Wilk normality test, and Kolmogorov-Smirnov test. Categorical variables were compared using Fisher’s exact test. A two-sided p value < 0.05 was considered statistically significant. Analyses were performed using GraphPad Prism 9.3.1 or RStudio 1.4.1717 with R 4.1.1. Between-cohort comparisons were unadjusted, and no multiplicity correction was applied. Because cohort status was completely linked to calendar period and etiologic composition, no multivariable model was used to estimate a disease-specific cohort effect.

## Results

### Patient cohort and patient characteristics

The study cohort (*n* = 600) comprised a C-19 cohort (*n* = 245) and an nC-19 cohort (*n* = 355), with all patients requiring vvECMO support due to respiratory failure (Fig. 1). Patient characteristics, including demographics, comorbidities and pre-ECMO parameters, are shown in Table 1. The etiologic composition of the nC-19 cohort is additionally summarized in Supplementary Table 1.

#### Comparison of patient characteristics and markers of disease severity

C-19 patients were older (57.0 years [IQR: 50.0–63.0] vs. 51.0 years [IQR: 39.0-62.5]), had a higher BMI (29.3 kg/m2 [IQR: 25.3–33.6] vs. 26.0 kg/m2 [IQR: 22.5–30.8]) and were more often male (74.7% vs. 66.8%) than nC-19 patients (Table 1). nC-19 patients were more frequently immunocompromised (27.0% vs. 6.5%), whereas C-19 patients were more likely to have rheumatologic disease (6.1% vs. 1.7%). At admission to the ARDS/ECMO center, C-19 patients had a lower Charlson Comorbidity Index (CCI) (2 [IQR: 1–3] vs. 3 [IQR: 1–5]) and a lower Sequential Organ Failure Assessment (SOFA) score (11 [IQR: 10–13] vs. 12 [IQR: 10–15]). APACHE II scores did not differ significantly between cohorts (Table 1). Septic shock was more frequent in nC-19 patients (65.4% vs. 13.9%), as was dialysis before vvECMO initiation (58.0% vs. 28.2%). nC-19 patients also had higher creatinine (1.90 mg/dl [IQR: 0.78–4.53] vs. 1.07 mg/dl [IQR: 0.73–1.91]), bilirubin (1.57 mg/dl [IQR: 0.88–2.53] vs. 0.73 mg/dl [IQR: 0.42–1.45]) and lactate levels (26.0 mg/dl [IQR: 15.0–59.0] vs. 10.0 mg/dl [IQR: 7.0–15.0]) before vvECMO initiation.

#### Comparison of respiratory support before vvECMO

Before vvECMO initiation, 86.9% of C-19 and 77.5% of nC-19 patients had undergone prone positioning, and 40.0% vs. 76.6% had received inhaled nitric oxide (NO) therapy (Table 1). These differences may have been influenced by era-related practice patterns. Respiratory rate (25/min [22–27] vs. 24/min [20–27]) and minute ventilation (9.6 L/min [7.9–11.6] vs. 9.4 L/min [7.5–11.8]) were similar. Peak airway pressure (pPeak; 35.0 mbar [31.0–38.0] vs. 40.0 mbar [36.0-44.9]), positive end-expiratory pressure (PEEP; 16.0 mbar [14.0–19.0] vs. 19.4 mbar [16.0–21.0]) and plateau airway pressure (31.0 mbar [28.0–34.0] vs. 36.0 mbar [33.1–40.8]) were significantly higher in nC-19 than in C-19 patients. C-19 patients had a higher Horovitz index (95.6 vs. 65.5), whereas arterial carbon dioxide partial pressure (PaCO2) did not differ significantly between cohorts (68.7 vs. 64.7 mmHg). Median time from intubation to cannulation was 8.0 days [IQR: 2.0–15.0] in C-19 and 3.1 days [IQR: 1.1–8.9] in nC-19 ARDS. C-19 patients were less often resuscitated before ECMO initiation (5.3% vs. 18.9%), required longer vvECMO support (26.0 days [IQR: 13.0–41.0] vs. 12.6 days [IQR: 5.9–25.6]) and had longer total MV duration (39.0 days [IQR: 24.8–59.0] vs. 24.7 days [IQR: 15.7–41.4]) and ICU stay. ECMO therapy was more often initiated externally by the mobile Charité ECMO retrieval team in C-19 patients (37.1% vs. 19.7%). In summary, nC-19 ARDS was more frequently associated with septic shock (65.4% vs. 13.9%) and multi-organ impairment, whereas C-19 patients more often presented with a pulmonary-predominant pattern of organ dysfunction at ECMO initiation. Better oxygenation and longer MV before vvECMO in C-19 were not accompanied by a statistically significant difference in ICU mortality (55.1% vs. 47.9%; Table 1).

#### Comparison of patient characteristics of C-19 survivors vs. non-survivors

Within the C-19 cohort, survivors had lower BMI, CCI, SOFA and APACHE II scores than non-survivors (Table 3). Survivors were ventilated with lower plateau airway pressures (31.0 mbar [28.8–33.0] vs. 32.0 mbar [28.0–35.0]) and lower PEEP values (15.0 mbar [14.0–18.0] vs. 17.0 mbar [15.0–20.0]), although the median differences of one and two millibars, respectively, are of questionable biological significance. Oxygenation was more impaired in non-survivors (Horovitz index 89.2 [IQR: 73.0-118.0]) than in survivors (110.0 [IQR: 83.6-149.2]). Notably, PaCO2 was higher in survivors (73.0 mmHg [IQR: 61.1–85.5]) than in non-survivors (64.2 mmHg [IQR: 56.1–74.4]), while minute ventilation was similar. Survivors had a lower incidence of septic shock (7.5% vs. 19.3%), dialysis before ECMO onset (16.4% vs. 37.8%), NO therapy (30.0% vs. 48.1%) and lower creatinine (0.86 mg/dl [0.60–1.37] vs. 1.5 mg/dl [0.9–2.3]), bilirubin (0.61 mg/dl [0.39–1.01] vs. 0.8 mg/dl [0.5–2.1]) and lactate levels (9.5 mg/dl [6.3–14.0] vs. 11.0 mg/dl [9.0–16.0]). Survivors required more days on ECMO (29.0 [18.0-42.8] vs. 23.0 [10.0–38.0]), longer MV (56.0 [40.8–74.0] vs. 30.0 [18.8–43.3]) and longer ICU treatment (47.0 [34.0–62.0] vs. 24.0 [11.5–39.5]), but fewer hours of dialysis (0.0 [0.0-224.0] vs. 148.0 [18.4–487.0]; Table 3). Days on MV before ECMO were comparable between groups.

**Table 3:**
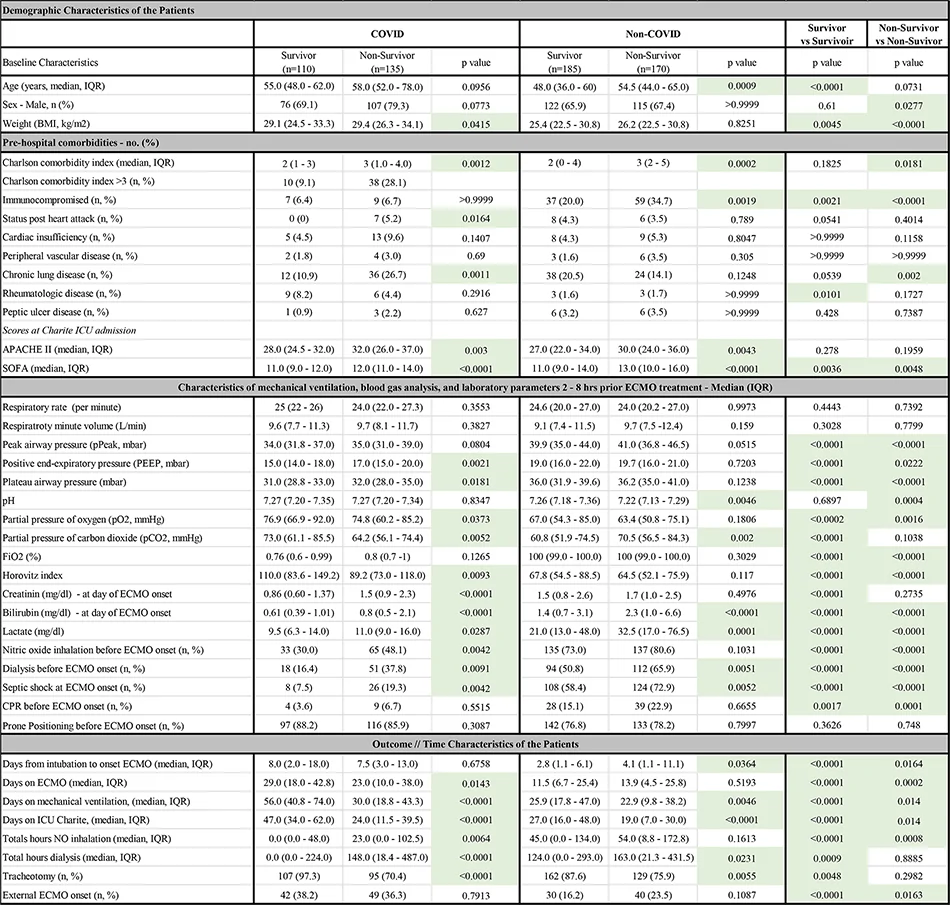
Characteristics of survivors vs. non-survivors of the COVID-19 and non-COVID-19 cohort

#### Comparison of nC-19 survivors vs. non-survivors

In nC-19 ARDS, factors associated with an unfavorable outcome included pre-existing comorbidity (CCI 2 [0–4] vs. 3 [2–5]), immunosuppression (20.0% vs. 34.7%), septic shock (58.4% vs. 72.9%) and dialysis before ECMO onset (50.8% vs. 65.9%) (Table 3). PaO2 did not differ between survivors and non-survivors; in contrast to C-19 survivors, nC-19 survivors had lower PaCO2 (60.8 mmHg [51.9–74.5] vs. 70.5 mmHg [56.5–84.3]). Airway pressures and minute ventilation were similar between survivors and non-survivors. Survivors had lower bilirubin (1.4 mg/dl [0.7–3.1] vs. 2.3 mg/dl [1.0-6.6]) and lactate levels (21.0 mg/dl [13.0–48.0] vs. 32.5 mg/dl [17.0-76.5]) than non-survivors. Days on MV before ECMO were shorter in survivors than in non-survivors (2.8 [1.1–6.1] vs. 4.1 [1.1–11.1]), whereas no such difference was observed in the C-19 cohort. Survivors required longer MV (25.9 [17.8–47.0] vs. 22.9 [9.8–38.2]) and ICU stay (27.0 [16.0–48.0] vs. 19.0 [7.0–30.0]) and fewer hours of dialysis (124.0 [0.0-293.0] vs. 163.0 [21.3-431.5]), whereas days on vvECMO were comparable. These findings indicate that survivor/non-survivor characteristics differed between C-19 and nC-19 ARDS.

#### Comparison of C-19 and nC-19 survivors

Compared with nC-19 survivors, C-19 survivors were older (55.0 [48.0–62.0] vs. 48.0 [36.0–60.0]), had a higher BMI (29.1 [24.5–33.3] vs. 25.4 [22.5–30.8]) and were less often immunocompromised (6.4% vs. 20.0%). C-19 survivors were ventilated with lower pPeak (34.0 mbar [31.8–37.0] vs. 39.9 mbar [35.0–44.0]) and PEEP (15.0 mbar [14.0–18.0] vs. 19.0 mbar [16.0–22.0]) and had better oxygenation (Horovitz index 110.0 [IQR: 83.6-149.2] vs. 67.8 [IQR: 54.5–88.5]). PaCO2 was significantly higher in C-19 survivors than in nC-19 survivors (73.0 mmHg [61.1–85.5] vs. 60.8 mmHg [51.9–74.5]). C-19 survivors had lower lactate (9.5 mg/dl [6.3–14.0] vs. 21.0 mg/dl [13.0–48.0]), bilirubin (0.61 mg/dl [0.39–1.01] vs. 1.4 mg/dl [0.7–3.1]) and creatinine levels (0.86 mg/dl [0.60–1.37] vs. 1.5 mg/dl [0.8–2.6]), lower rates of septic shock (7.5% vs. 58.4%) and less need for renal replacement therapy (16.4% vs. 50.8%). Duration of MV before ECMO was longer in C-19 survivors (8.0 [2.0–18.0] vs. 2.8 [1.1–6.1]), and ECMO was more often initiated externally (38.2% vs. 16.2%). C-19 survivors had more days on MV (56.0 [40.8–74.0] vs. 25.9 [17.8–47.0]), more days on ECMO (29.0 [18.0-42.8] vs. 11.5 [6.7–25.4]), longer ICU stay (47.0 [34.0–62.0] vs. 27.0 [16.0–48.0]) and more frequent tracheostomy (97.3% vs. 87.6%).

#### Comparison of C-19 and nC-19 non-survivors

Compared with nC-19 non-survivors, C-19 non-survivors had a lower CCI distribution (3 [1.0–4.0] vs. 3 [2–5]), higher BMI (29.4 [26.3–34.1] vs. 26.2 [22.5–30.8]), lower SOFA score (12.0 [11.0–14.0] vs. 13.0 [10.0–16.0]), were more often male (79.3% vs. 67.4%), were less often immunocompromised (6.7% vs. 34.7%) and more frequently had chronic lung disease (26.7% vs. 14.1%). C-19 non-survivors were ventilated with lower pPeak (35.0 mbar [31.0–39.0] vs. 41.0 mbar [36.8–46.5]), PEEP (17.0 mbar [15.0–20.0] vs. 19.7 mbar [16.0–21.0]) and plateau pressure (32.0 mbar [28.0–35.0] vs. 36.2 mbar [35.0–41.0]). PaCO2 did not differ between groups, whereas PaO2 was higher in C-19 non-survivors (74.8 mmHg [60.2–85.2] vs. 63.4 mmHg [50.8–75.1]) and exceeded the median PaO2 observed in nC-19 survivors (Table 3). C-19 non-survivors had lower rates of septic shock (19.3% vs. 72.9%), dialysis before ECMO onset (37.8% vs. 65.9%) and CPR before ECMO onset (6.7% vs. 22.9%), and lower lactate (11.0 mg/dl [9.0–16.0] vs. 32.5 mg/dl [17.0-76.5]) and bilirubin (0.8 mg/dl [0.5–2.1] vs. 2.3 mg/dl [1.0-6.6]). C-19 non-survivors received more days of vvECMO (23.0 [10.0–38.0] vs. 13.9 [4.5–25.8]), longer MV (30.0 [18.8–43.3] vs. 22.9 [9.8–38.2]) and longer ICU treatment (24.0 [11.5–39.5] vs. 19.0 [7.0–30.0]). They were ventilated more days before ECMO than nC-19 non-survivors (7.5 [3.0–13.0] vs. 4.1 [1.1–11.1]), and ECMO was more often initiated externally (36.3% vs. 23.5%). Taken together, C-19 patients with severe ARDS receiving vvECMO differed significantly from nC-19 ARDS patients. Multi-organ impairment was less pronounced in C-19 patients. Despite this descriptive difference, this unadjusted difference does not finally prove a disease-specific phenotype or causal mortality effect.

### RESP score

The RESP Score predicts the in-hospital survival of adult patients undergoing vvECMO support for respiratory failure using pre-ECMO clinical parameters as input variables [6]. To investigate how well the RESP score (Supplementary Table 4) predicts survival in the C-19 and nC-19 cohorts, the primary RESP analysis included patients with all remaining score inputs available after the predefined zero coding (C-19: *n* = 147; nC-19: *n* = 229; Fig. 2A; Supplementary Table 5A and B). Data on neuromuscular blocking agents were not available in the nC-19 cohort, whereas data on bicarbonate therapy before ECMO initiation were unavailable for both cohorts. Observed survival by RESP risk class is displayed in Fig. 2 alongside the survival proportions reported for the original RESP classes. Multivariable logistic regression analysis was performed to assess whether RESP score pre-ECMO parameters were associated with survival. Examination revealed an AUC of 0.73 in the non-COVID cohort (Fig. 2B) and of 0.60 in the C-19 cohort (Fig. 2C). Thus, the RESP score demonstrated a predictive ability comparable to previous findings, while the RESP score showed limited discrimination in C-19 patients of our cohort.

A study by Schmidt et al. [6] reported the RESP score AUC for viral pneumonia groups to be 0.73 (95% CI: 0.65–0.80). Similar to Schmidt et al.’s findings, we found age, immunosuppression, and duration of MV before ECMO related to survival in the nC-19 cohort. In contrast to their findings, central nervous system dysfunction, acute associated (non-pulmonary) infection, cardiac arrest before ECMO, PaCO2 (≥ 75 mmHg), and Peak inspiratory pressure (≥ 42 mbar) were not associated with survival in our nC-19 cohort (Supplementary Table 6). None of the RESP score variables showed a statistically significant association with survival in the C-19 cohort (Supplementary Table 6).

### Machine learning and mortality prediction

**Fig. 4:**
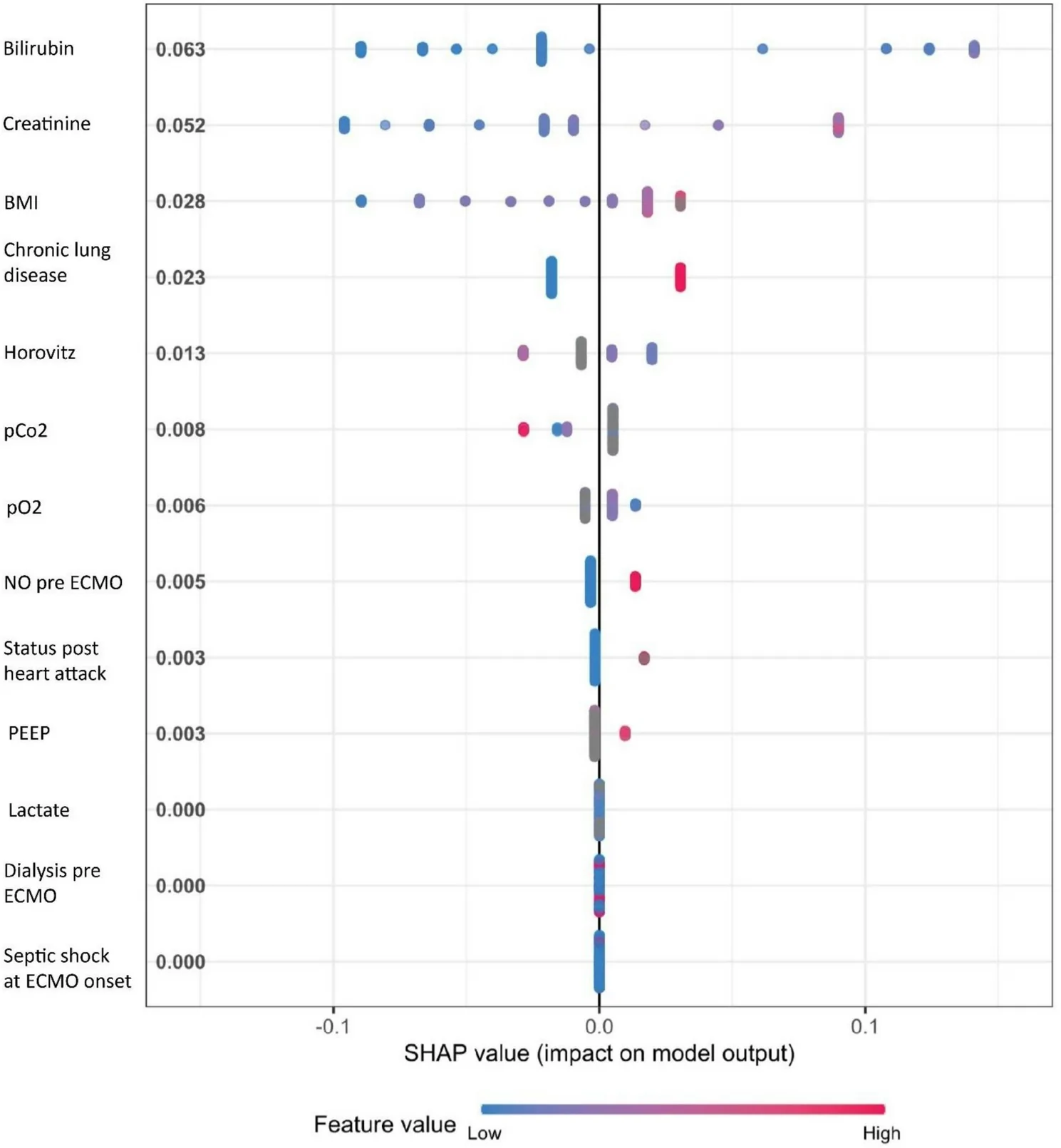
SHAP (Shapley Additive exPlanations) of the exploratory XGBoost model. Parameters on the y-axis are ranked in order of the average (absolute) SHAP value, indicating each parameter’s importance for the classification model. Individual data points (dots) represent the model’s SHAP value per parameter for each patient. The position of a dot on the x-axis represents the impact of the observed parameter value on the model’s output for the individual patient. Positive SHAP values indicate the effect of predicting ICU mortality, and negative SHAP values indicate the impact of predicting survival. The color of the individual patient’s dots (Feature value) represents the value of the observed parameters, with blue indicating lower and red higher values (e.g., red indicates high creatinine value), or for categorical variables, the absence/presence (e.g., chronic lung disease: blue: no chronic lung disease, red: lung disease). SHAP values quantify model attribution and do not imply causal effects

As RESP showed limited discrimination in C-19 patients, we aimed at identifying parameters associated with mortality in C-19 vvECMO patients. We therefore implemented a machine learning classifier predicting mortality in vvECMO C-19 patients in a first step. Next, we calculated SHAP (SHapley Additive exPlanations) values to quantify feature contributions to the model output (Fig. 4). In a third step, we used this information to develop an exploratory C-19 vvECMO Mortality Prediction (CEMP) score.

The XGBoost classifier correctly classified 43 of 49 test-set cases (87.8%; exact binomial 95% CI 75.2–95.4%). The calculated SHAP values are presented in Fig. 4; they show how each feature contributes to the model output but do not imply causal effects. The four features with the greatest impact on C-19 outcome prediction (y-axis) were bilirubin, creatinine, body mass index (BMI), and chronic lung disease (Fig. 4). Individual patient values suggested that high bilirubin and high creatinine values contributed most to predicting ICU mortality. On the other hand, a low BMI contributed to predictions of survival. Chronic lung disease had a greater impact on the model output than its absence (as indicated by the dots on the x-axis). Based on this observation, four variables were selected for the exploratory CEMP score: BMI < 25, chronic lung disease, bilirubin > 3 mg/dl, and creatinine > 2 mg/dl, with integer weights based on the observed SHAP distributions (Table 4). These thresholds and weights were not prespecified and may overfit this cohort. Although the PaO₂/FiO₂ ratio and PaCO₂ ranked fifth and sixth, respectively, they were not included because of lower model attribution, substantial missingness, and dependence on ventilatory management.

**Table 4:**
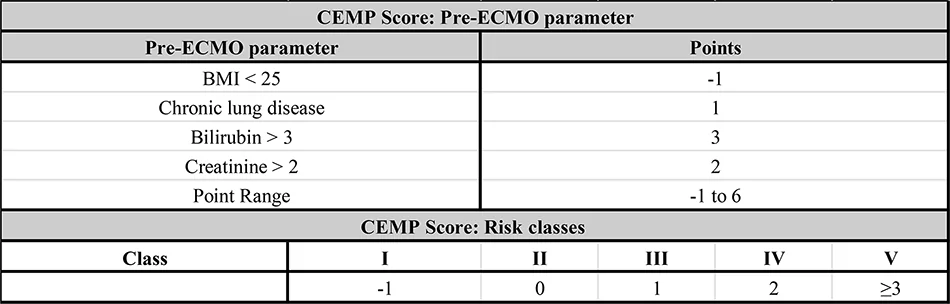
Components and risk classes of the exploratory COVID-19 ECMO Mortality Prediction (CEMP) score. The score ranges from -1 to 6 points and classifies patients into five risk classes (I–V). Cutoffs and integer weights were derived from the observed SHAP distributions and were not prespecified. Associations of the four score components with ICU mortality were estimated in the same complete-case cohort (Table 2); this does not constitute validation

The CEMP score ranges from − 1 to 6 points (Table 1) and is divided into five risk classes (Table 1), I (-1), II (0), III (1), IV (2), and V (≥ 3). ICU mortality per risk class is displayed in Fig. 3A for the 210 complete cases.

In addition, we calculated individual mortality rates of the score parameters. Fifty patients had a BMI below 25, with a mortality rate of 36%. In the same complete-case cohort, BMI below 25 was associated with lower mortality. Conversely, 36 patients out of 48 with chronic lung disease died (75%), 27 patients out of 30 with a bilirubin value over 3 died (90%), and 46 of 57 patients meeting the creatinine component threshold died (80.7%).

An exploratory multivariable analysis of the four CEMP components was conducted in the same complete-case cohort; the apparent AUC was 0.757 (95% CI 0.69–0.82) (Fig. 3B). Moreover, odds ratios, 95% confidence intervals, and p-values are listed in Table 1. Results indicate that all four parameters were associated with mortality in this cohort. This analysis does not constitute internal or external validation.

## Discussion

The main strength of this study is the availability of a large, highly granular single-center vvECMO cohort that enabled direct comparison of C-19 and nC-19 ARDS. This comparative framework revealed differences in organ dysfunction burden, respiratory characteristics, and treatment duration, while ICU mortality did not differ significantly between groups. In addition, our findings indicate that prognostic tools established in mixed-etiology ARDS may not transfer unchanged to COVID-19, which motivated the derivation of the CEMP score.

Overall patient characteristics and data availability for both cohorts allowed for clinical comparison. We based our calculations on a C-19 cohort recruited within a specialized ARDS/ECMO center over 23 months during the pandemic between March 1st, 2020, and January 31st, 2022. The cohort comprised patients infected with the wild-type, the alpha, beta, or delta variant. Patients were referred from hospitals in Berlin and the surrounding regions within a network, which is comparable with those from the Greater Paris region as previously published by Lebreton et al. [11]. Data from the Extracorporeal Life Support Organization (ELSO) Registry, Greater Paris, Germany, and Chile showed mortality rates between 39% and 74% [12–16] for this patient collective. Previous findings in a German cohort reported a 62–80% mortality on vvECMO [3], depending on the center´s experience, while our cohort has a mortality rate of 55.1%. Generally, survival was found to depend on multiple factors, including inclusion criteria themselves, as well as local resource allocation, timing of ECMO initiation, and experience of the center [3, 11]. Notably, our ARDS/ECMO center never faced strain, even with a significant patient volume, and the overall ICU capacities across our network consistently met demand. No pandemic-specific capacity restrictions were applied; each patient was assessed individually by ARDS/ECMO specialists for eligibility. Although this is a single-center cohort, the number of patients in both cohorts is almost as high as the combined cohort from 26 other high-volume centers in Germany (*n* = 673 C-19 ARDS vvECMO patients) [3], hence classifying the Charité ARDS/ECMO Center as a high-volume ECMO center.

Direct comparative studies have reported heterogeneous outcomes. Raasveld et al. found no significant difference in 28-day mortality between C-19 and historical nC-19 ECMO cohorts [7], as did Autschbach et al. for 90-day mortality after propensity matching [17]. In the multicenter C-19/H1N1 comparison by Fanelli et al., higher crude 60-day mortality in C-19 was no longer significant after adjustment [8]. Conversely, the ELSO analysis by Chandel et al. found higher adjusted hospital mortality in C-19 than in other viral ARDS [9]; large US and South Korean studies also reported higher mortality, although the latter assessed longer-term outcomes [18, 19].

Smaller mixed-etiology studies likewise illustrate variation in patient characteristics and outcomes [20, 21]; a meta-analysis of 24 comparative studies found lower survival in C-19 [22]. Differences in comparator etiology, case mix, ECMO selection, and outcome horizon limit direct comparisons. In our cohort, older age and higher BMI in C-19 contrasted with more immunosuppression, shock, and extrapulmonary organ dysfunction in nC-19. These differences may influence unadjusted mortality, and the non-significant result does not establish equivalence. Treatment era is also relevant: COVID-only ELSO data showed changing mortality during 2020 [23], reinforcing the limitations of historical comparisons.

The nC-19 cohort is one of the largest single-center nC-19 vvECMO cohorts and has been previously analyzed in multiple contexts [24–26]. Patients with acute pancreatitis and secondary sepsis were excluded because their underlying conditions and treatment could substantially affect outcome and reduce comparability with the C-19 cohort. The nC-19 cohort was recruited from January 2007 to December 2018, whereas the C-19 cohort was recruited during 2020–2022. During this interval, referral pathways, ECMO candidacy, ventilatory and prone-positioning practices, adjunctive therapies, retrieval-team availability, and general ICU care may have changed. The higher frequency of external ECMO initiation in C-19 also reflects a changed referral and retrieval environment. Because cohort status and treatment era were fully linked, their effects cannot be separated. Notably, the recruitment of a contemporaneous representative nC-19 cohort during the pandemic was not feasible because nC-19 ARDS incidence at our center was very low. The nC-19 cohort comprised multiple ARDS etiologies; this heterogeneity should be considered when interpreting direct comparisons. However, this heterogeneity reflects the mixed-etiology ARDS populations recruited in major studies and for which tools such as RESP were developed and is therefore relevant to assessing transferability across ARDS populations. We did not perform post hoc outcome analyses across etiologic strata because these analyses were not prespecified and several strata were small.

The baseline characteristics of patients before cannulation differed significantly. C-19 ARDS patients were older, had a higher BMI, had a lower CCI, and were less frequently immunocompromised, whereas nC-19 patients more often exhibited multi-organ impairment and shock. Septic shock and extrapulmonary organ impairment were more frequent in C-19 non-survivors than in survivors. In contrast, a smaller overall proportion of C-19 patients had multi-organ impairment and septic shock at ECMO initiation than nC-19 patients. A possible explanation is the primary respiratory tropism of SARS-CoV-2, although COVID-19 can involve multiple organ systems [27, 28]. Secondary bacterial infection may have contributed to septic shock in the rather small proportion of shock at ECMO initiation in the C-19 cohort. In nC-19 ARDS, bacterial infection with a suspected pulmonary source was the most frequent etiology in our cohort, while 19.7% had a non-SARS-CoV-2 viral infection. Overall, C-19 patients had less extrapulmonary organ dysfunction at ECMO initiation.

Both cohorts demonstrated severely impaired gas exchange, but C-19 patients had less severe oxygenation failure. Notably, oxygenation in C-19 non-survivors was even better than in nC-19 survivors. C-19 survivors had higher PaCO₂ than C-19 non-survivors. In contrast, nC-19 survivors had lower PaCO₂ than non-survivors, while respiratory minute volume and respiratory frequency were similar across subgroups.

The higher PaCO₂ observed in C-19 survivors might be attributable, at least in part, to a restrictive, fibroproliferative ARDS phenotype previously described in late severe C-19 ARDS [2]. However, this interpretation remains speculative because the present analysis did not include histopathology, systematic imaging analysis, or respiratory-system compliance and therefore cannot ultimately prove this phenotype in the current cohort.

Renal replacement therapy was significantly more often required in the nC-19 cohort. In both cohorts, renal replacement therapy was required significantly more often before vvECMO therapy in non-survivors. Independent of vvECMO therapy, kidney failure is a marker of disease severity and is associated with increased in-hospital mortality [26, 29, 30]. Accordingly, the need for renal replacement therapy was associated with mortality in this cohort for both C-19 and nC-19 patients.

Overall, C-19 patients required more ICU-related resources than nC-19 patients, such as significantly prolonged MV and vvECMO treatment time, as well as a prolonged stay in the ICU. This is consistent with previous findings, where C-19 ARDS has been associated with longer MV and vvECMO treatment compared to H1N1-related ARDS or ARDS of other etiologies [1, 8, 9, 20].

Prognostic assessment before ECMO initiation is clinically relevant, especially in the context of limited resources and the need to avoid “burdensome” therapies for patients.

The observed cohort differences and limited RESP discrimination motivated exploratory evaluation of alternative parameters.

We developed an exploratory C-19 vvECMO mortality prediction (CEMP) score using an explainable machine-learning approach and SHAP as a data-driven feature-ranking method. In this cohort, CEMP had a higher apparent AUC than RESP. However, the analyses were not designed as a formal head-to-head comparison, and the estimate may be optimistic. Given the sample size, missingness of candidate variables, and lack of external validation, CEMP should be interpreted as a cohort-derived tool requiring independent validation rather than as a replacement for existing prognostic approaches. The four CEMP components are less directly determined by ventilator settings, but their selection may reflect missingness of respiratory variables and does not imply that respiratory parameters are unimportant. Missing values, particularly for arterial blood gas and mechanical ventilation variables in patients with external vvECMO initiation, limit the explanatory power of these variables. Moreover, the original RESP score was developed using 2355 patient cases [6], whereas our model training used 196 cases. Together with the absence of patients infected with the Omicron variant and the lack of external validation, these factors necessitate further testing of CEMP. Finally, the proposed CEMP score requires independent validation. In addition, CEMP may potentially be combined with novel prognostic parameters from previously published proteomic approaches [31, 32]. Finally, the single-center design of our study with its aforementioned limitations does not establish general inadequacy of RESP for C-19 ARDS patients.

## Conclusion

In this large single-center comparative vvECMO cohort, C-19 and nC-19 ARDS differed substantially in pre-ECMO characteristics, organ dysfunction burden, and treatment duration, while ICU mortality did not differ significantly. The higher PaCO₂ observed among C-19 survivors may reflect a restrictive phenotype, but this unadjusted association does not provide proof of a restrictive or fibroproliferative phenotype. Observed differences should be interpreted in light of etiology, case mix, referral, selection, and treatment era. In this cohort, RESP showed limited discrimination in C-19, whereas CEMP showed higher apparent discrimination within the derivation cohort but remains exploratory and requires calibration and independent external validation before clinical application.

## Electronic Supplementary material

Below is the link to the electronic supplementary material.

Supplementary Material

## Data Availability

The datasets generated and analyzed for this study are not publicly available and cannot be shared with third parties due to institutional data protection policies and restrictions imposed by Charité – Universitätsmedizin Berlin. No individual-level data can be made available. Further information regarding the study may be obtained from the corresponding author upon reasonable request.

## Abbreviations

APACHE II: Acute Physiology and Chronic Health Evaluation II
ARDS: Acute Respiratory Distress Syndrome
AUC: Area Under the Curve
BMI: Body Mass Index
CCI: Charlson Comorbidity Index
CEMP: COVID–19 ECMO Mortality Prediction Score
COVID: 19 (C–19)–Coronavirus Disease 2019
CPR: Cardiopulmonary Resuscitation
ECMO: Extracorporeal Membrane Oxygenation
ELSO: Extracorporeal Life Support Organization
FiO₂: Fraction of Inspired Oxygen
ICU: Intensive Care Unit
IQR: Interquartile Range
MV: Mechanical Ventilation
nC: 19–Non–COVID–19
NO: Nitric Oxide
PaCO₂: Arterial Partial Pressure of Carbon Dioxide
PaO₂: Arterial Partial Pressure of Oxygen
PCR: Polymerase Chain Reaction
PEEP: Positive End–Expiratory Pressure
pPeak: Peak Airway Pressure
RESP: Respiratory ECMO Survival Prediction
ROC: Receiver Operating Characteristic
SHAP: SHapley Additive exPlanations
SOFA: Sequential Organ Failure Assessment
SARS: CoV–2–Severe Acute Respiratory Syndrome Coronavirus 2
vaECMO: Veno–Arterial Extracorporeal Membrane Oxygenation
vvaECMO: Veno–Veno–Arterial Extracorporeal Membrane Oxygenation
vvECMO: Veno–Venous Extracorporeal Membrane Oxygenation
XGBoost: Extreme Gradient Boosting

## References

[1] Russ M, et al. COVID-19 Patients Require Prolonged Extracorporeal Membrane Oxygenation Support for Survival Compared With Non-COVID-19 Patients. Crit Care Explor. 2022;4:e0671. 10.1097/CCE.0000000000000671 PMC8966959 35372842

[2] Wendisch D, et al. SARS-CoV-2 infection triggers profibrotic macrophage responses and lung fibrosis. Cell. 2021;184:6243–e626127. 10.1016/j.cell.2021.11.033 PMC8626230 34914922

[3] Herrmann J, et al. Key characteristics impacting survival of COVID-19 extracorporeal membrane oxygenation. Crit Care. 2022;26:190. 10.1186/s13054-022-04053-6 PMC9238175 35765102

[4] Calfee CS, et al. Subphenotypes in acute respiratory distress syndrome: latent class analysis of data from two randomised controlled trials. Lancet Respir Med. 2014;2:611–20. 10.1016/S2213-2600(14)70097-9 PMC4154544 24853585

[5] Zhang J, et al. Phenotypes of severe COVID-19 ARDS receiving extracorporeal membrane oxygenation. Br J Anaesth. 2021;126:e130–2. 10.1016/j.bja.2020.12.023 PMC7836959 33487454

[6] Schmidt M, et al. Predicting survival after extracorporeal membrane oxygenation for severe acute respiratory failure. The Respiratory Extracorporeal Membrane Oxygenation Survival Prediction (RESP) score. Am J Respir Crit Care Med. 2014;189:1374–82. 10.1164/rccm.201311-2023OC 24693864

[7] Raasveld SJ, et al. Extracorporeal Membrane Oxygenation in Patients With COVID-19: An International Multicenter Cohort Study. J Intensive Care Med. 2021;36:910–7. 10.1177/08850666211007063. PMC8267077 33823709

[8] Fanelli V, et al. Extracorporeal membrane oxygenation for COVID-19 and influenza H1N1 associated acute respiratory distress syndrome: a multicenter retrospective cohort study. Crit Care. 2022;26:34. 10.1186/s13054-022-03906-4. PMC8817653 35123562

[9] Chandel A, et al. Extracorporeal Membrane Oxygenation for COVID-19: Comparison of Outcomes to Non-COVID-19-Related Viral Acute Respiratory Distress Syndrome From the Extracorporeal Life Support Organization Registry. Crit Care Explor. 2023;5:e0861. 10.1097/CCE.0000000000000861. PMC9901999 36760815

[10] Rubenfeld GD, et al. Incidence and outcomes of acute lung injury. N Engl J Med. 2005;353:1685–93. 10.1056/NEJMoa050333 16236739

[11] Lebreton G, et al. Extracorporeal membrane oxygenation network organisation and clinical outcomes during the COVID-19 pandemic in Greater Paris, France: a multicentre cohort study. Lancet Respir Med. 2021;9:851–62. 10.1016/S2213-2600(21)00096-5 PMC8055207 33887246

[12] Barbaro RP, et al. Extracorporeal membrane oxygenation support in COVID-19: an international cohort study of the Extracorporeal Life Support Organization registry. Lancet. 2020;396:1071–8. 10.1016/S0140-6736(20)32008-0 PMC7518880 32987008

[13] Friedrichson B, et al. Extracorporeal membrane oxygenation in coronavirus disease 2019: A nationwide cohort analysis of 4279 runs from Germany. Eur J Anaesthesiol. 2022;39:445–51. 10.1097/EJA.0000000000001670 35180152

[14] Karagiannidis C, et al. High In-Hospital Mortality Rate in Patients with COVID-19 Receiving Extracorporeal Membrane Oxygenation in Germany: A Critical Analysis. Am J Respir Crit Care Med. 2021;204:991–4. 10.1164/rccm.202105-1145LE PMC8534613 34283685

[15] Diaz RA, et al. Extracorporeal Membrane Oxygenation for COVID-19-associated Severe Acute Respiratory Distress Syndrome in Chile: A Nationwide Incidence and Cohort Study. Am J Respir Crit Care Med. 2021;204:34–43. 10.1164/rccm.202011-4166OC PMC8437120 33823118

[16] Karagiannidis C, et al. In-hospital mortality, comorbidities, and costs of one million mechanically ventilated patients in Germany: a nationwide observational study before, during, and after the COVID-19 pandemic. Lancet Reg Health Eur. 2024;42:100954. 10.1016/j.lanepe.2024.100954 PMC11281923 39070745

[17] Autschbach T, et al. Outcomes of Extracorporeal Membrane Oxygenation for Acute Respiratory Distress Syndrome in COVID-19 Patients: A Propensity-Matched Analysis. J Clin Med. 2021;10:2547. 10.3390/jcm10122547. PMC8227073 34207573

[18] Ruhi-Williams P, et al. Analysis of Veno-Venous Extracorporeal Membrane Oxygenation for COVID-19 Compared to Non-COVID Etiologies. Ann Surg. 2023;278:464–70. 10.1097/SLA.0000000000005959. 37325899

[19] Oh TK, Song IA. Extracorporeal membrane oxygenation support in adult patients: Comparing COVID-19- and non-COVID-19-associated respiratory failure. Perfusion. 2025;40:1137–44. 10.1177/02676591241281792. 39222402

[20] Yaqoob H, et al. Extracorporeal membrane oxygenation in COVID-19 compared to other etiologies of acute respiratory failure: A single-center experience. Heart Lung. 2023;57:243–9. 10.1016/j.hrtlng.2022.10.003. PMC9582301 36274533

[21] Mirus M, et al. Effects of the primary lung infection on outcomes in patients with severe ARDS treated with ECMO: a retrospective analysis. Front Med. 2025;12:1662239. 10.3389/fmed.2025.1662239. PMC12626973 41267858

[22] Bertini P, Marabotti A, Sangalli F, Paternoster G. Survival difference in patients treated with extracorporeal membrane oxygenation in COVID-19 vs. non-COVID ARDS: a systematic review and meta-analysis. Minerva Anestesiol. 2024;90:1139–50. 10.23736/S0375-9393.24.18219-3. 39630142

[23] Barbaro RP, et al. Extracorporeal membrane oxygenation for COVID-19: evolving outcomes from the international Extracorporeal Life Support Organization Registry. Lancet. 2021;398:1230–8. 10.1016/S0140-6736(21)01960-7. PMC8480964 34599878

[24] Bünger V, et al. Potential of cell-free hemoglobin and haptoglobin as prognostic markers in patients with ARDS and treatment with veno-venous ECMO. J Intensive Care. 2023;11:15. 10.1186/s40560-023-00664-5 PMC10116665 37081577

[25] Hunsicker O, et al. Timing, Outcome, and Risk Factors of Intracranial Hemorrhage in Acute Respiratory Distress Syndrome Patients During Venovenous Extracorporeal Membrane Oxygenation. Crit Care Med. 2021;49:e120–9. 10.1097/CCM.0000000000004762 33323749

[26] Graw JA, et al. The role of cell-free hemoglobin and haptoglobin in acute kidney injury in critically ill adults with ARDS and therapy with VV ECMO. Crit Care. 2022;26:50. 10.1186/s13054-022-03894-5 PMC8864920 35193645

[27] Gupta A, et al. Extrapulmonary manifestations of COVID-19. Nat Med. 2020;26:1017–32. 10.1038/s41591-020-0968-3 PMC11972613 32651579

[28] Jain U. Effect of COVID-19 on the Organs. Cureus. 2020;12:e9540. 10.7759/cureus.9540 PMC7470660 32905500

[29] Ronco C, Reis T, Husain-Syed F. Management of acute kidney injury in patients with COVID-19. Lancet Respir Med. 2020;8:738–42. 10.1016/S2213-2600(20)30229-0 PMC7255232 32416769

[30] Cheng Y, et al. Kidney disease is associated with in-hospital death of patients with COVID-19. Kidney Int. 2020;97:829–38. 10.1016/j.kint.2020.03.005 PMC7110296 32247631

[31] Demichev V, et al. A proteomic survival predictor for COVID-19 patients in intensive care. PLOS Digit Health. 2022;1:e0000007. 10.1371/journal.pdig.0000007 PMC9931303 36812516

[32] Hartl J, et al. Quantitative protein biomarker panels: a path to improved clinical practice through proteomics. EMBO Mol Med. 2023;15:e16061. 10.15252/emmm.202216061 PMC10086577 36939029

