# Supplementary material for: Pre-ECMO characteristics, organ dysfunction and exploratory mortality prediction in COVID-19 versus non-COVID-19 ARDS treated with veno-venous ECMO: a retrospective cohort study
Source: BMC Anesthesiol. 2026 Oct 7;26:601. doi: 10.1186/s12871-026-04306-9 (PMC13643991; doi:10.1186/s12871-026-04306-9)
Supplement: Supplementary file 1 [file 12871_2026_4306_MOESM1_ESM.docx]

**Supplementary material**

| **Etiology** | **n** | **%** |
| --- | --- | --- |
| Bacterial | 120 | 33.8 |
| Other viral (non-SARS-CoV-2) | 70 | 19.7 |
| Aspiration | 45 | 12.7 |
| Fungal | 44 | 12.4 |
| Mixed | 30 | 8.5 |
| Unknown | 46 | 12.9 |
| **Total** | **355** | **100.0** |

**Supplementary Table 1:** Etiologic composition of the non-COVID-19 ARDS cohort

**Supplementary Table 2**: Unavailable data points for all patients grouped by cohort. Data points were either not measured or not documented and thus unavailable.

**Supplementary Table 3**: Not available data points of survivors and non-survivors of the COVID-19 and non-COVID-19 cohorts. Data points were either not measured or not documented and thus unavailable.

| Age (years) | 18-49 | **0** |
| --- | --- | --- |
|  | 50-59 | **−2** |
|  | ≥60 | **−3** |
| Immunocompromised status | | **−2** |
| Mechanical ventilation before initiation of ECMO | <48 hours | **3** |
|  | 48 hours to 7 days | **1** |
|  | >7 days | **0** |
| Acute respiratory diagnosis group (select only one) | Viral pneumonia | **3** |
|  | Bacterial pneumonia | **3** |
|  | Asthma | **11** |
|  | Trauma and burn | **3** |
|  | Aspiration pneumonitis | **5** |
|  | Other acute respiratory diagnoses | **1** |
|  | Nonrespiratory and chronic respiratory diagnoses | **0** |
| Central nervous system dysfunction | | **−7** |
| Acute-associated (nonpulmonary) infection | | **−3** |
| Neuromuscular blockade before ECMO | | **1** |
| Nitric oxide use before ECMO | | **−1** |
| Bicarbonate infusion before ECMO | | **−2** |
| Cardiac arrest before ECMO | | **−2** |
| P_aCO2_ (mmHg) | <75 | **0** |
|  | ≥75 | **−1** |
| Peak inspiratory pressure (cmH_2_O) | <42 | **0** |
|  | ≥42 | **−1** |
| **Total Score** |  | **-22 to +15** |
|  |  |  |
| **Hospital Survival by Risk Class** | | |
| **Risk Class** | Survival Rate | **Score** |
| **I** | 92% | **≥6** |
| **II** | 76% | **3 to 5** |
| **III** | 57% | **-1 to 2** |
| **IV** | 33% | **-5 to -2** |
| **V** | 18% | **≤ -6** |

**Supplementary Table 4**:

Definition and calculation of RESP score. Modified from Schmidt et al. ^6^

| **A. Non-COVID-19: complete RESP-score data (n=229)** | | | | | | |
| --- | --- | --- | --- | --- | --- | --- |
| **Class** | **I** | **II** | **III** | **IV** | **V** | **All** |
| n | 2 | 16 | 72 | 94 | 45 | 229 |
| Deaths, n | 0 | 2 | 28 | 48 | 29 | 107 |
| **Observed survival, %** | **100.0** | **87.5** | **61.1** | **48.9** | **35.6** | **53.3** |
| Original RESP survival, % | 92 | 76 | 57 | 33 | 18 | — |
|  | | | | | | |
| **B. COVID-19: complete RESP-score data (n=147)** | | | | | | |
| **Class** | **I** | **II** | **III** | **IV** | **V** | **All** |
| n | 14 | 36 | 67 | 28 | 2 | 147 |
| Deaths, n | 10 | 19 | 36 | 18 | 2 | 85 |
| **Observed survival, %** | **28.6** | **47.2** | **46.3** | **35.7** | **0.0** | **42.2** |
| Original RESP survival, % | 92 | 76 | 57 | 33 | 18 | — |

**Supplementary Table 5**: Observed survival by RESP risk class in patients with all remaining score inputs available after predefined zero coding of systematically unavailable items: A, non-COVID-19 (n=229); B, COVID-19 (n=147). The “Original RESP survival” row reports the survival proportions published for the RESP risk classes. Zero coding is not equivalent to documented absence and may bias score distributions and performance estimates.

**Supplementary Table 6**: RESP score parameters associated with survival in multivariable analysis of the COVID-19 cohort (top) and non-COVID-19 cohort (bottom); ns: not statistically significant.

**Supplementary Methods:**

**Veno-venous ECMO availability, indication, and external vvECMO initiation**

All patients were treated at the ARDS-ECMO Center of the Charité-Universitätsmedizin Berlin, a tertiary referral center for ARDS and ECMO therapy for the Berlin and Brandenburg region. The interdisciplinary ARDS/ECMO center currently has the capacity for 54 ECMO patients. Before vvECMO initiation, an expert panel consisting of 3 intensivists discusses the indication for vvECMO support due to respiratory failure. In contrast to ICU/ECMO capacity in other countries, the vvECMO capacity of our center was at no time overloaded during the COVID-19 pandemic. Hospitals in the Berlin-Brandenburg region presented patients who developed severe ARDS to experts of the Charité ARDS-ECMO center.  Patients who were likely to require a vvECMO treatment immediately and/or for whom it was presumed that they would not survive transport without vvECMO to the Charité ARDS-ECMO Center were picked up by Charité vvECMO experts (with an ambulance or helicopter, depending on the distance). If required, vvECMO was initiated by our mobile teams in an external hospital, followed by immediate transport to Charité (Table 1; external ECMO onset). Available pre-ECMO respiratory data included respiratory rate, minute ventilation, peak airway pressure, PEEP, plateau pressure, FiO₂, arterial blood gases, prone positioning, and inhaled nitric oxide. Protocol adherence, tidal volume indexed to predicted body weight, driving pressure, and respiratory-system compliance were not consistently documented across both periods; neuromuscular blockade data were unavailable in the nC-19 cohort.

**Machine Learning and C-19 ECMO Mortality Score Development**

A machine learning-based workflow was implemented due to the limited discrimination of the RESP score in COVID-19 patients.

Classification: In the first step, a classifier was implemented to predict mortality. An XGBoost classifier, a gradient-boosted decision-tree ensemble, was selected. XGBoost classification was implemented using the R (R version 4.1.1 (2021-08-10) -- "Kick Things") XGBoost package, and as we aimed to solve a binary classification task, the “binary:logistic” configuration with a loss function for predicting probabilities for binary classification problems was implemented. Hyperparameter optimization was performed using a sequential grid-search strategy implemented with the caret package and three-fold cross-validation. The initial tuning grid evaluated the number of boosting rounds (nrounds, 1–50), learning rate (eta, 0.025, 0.05, 0.1, and 0.3), and maximum tree depth (max_depth, 1–5). Subsequent tuning steps refined min_child_weight, colsample_bytree, subsample, gamma, and eta. SHAP values were calculated from the training data.

Identification of variables: Candidate variables were derived from pre-ECMO parameters that differed between survivors and non-survivors in the overall C-19 cohort (Table 3). Composite scores (Charlson Comorbidity Index, SOFA, and APACHE II) were excluded to avoid incorporating overlapping variables. In addition, plateau airway pressure was not included in the data set, as values were missing in 151 out of 245 cases. Thus, the final data set consisted of thirteen parameters: bilirubin, creatinine, BMI, chronic lung disease, PaO₂/FiO₂ ratio, PaCO₂, PaO₂, pre-ECMO nitric oxide, history of myocardial infarction, PEEP, lactate, dialysis before ECMO, and septic shock at ECMO initiation. The dataset included n=245 patients and was randomly split into a training (n=196) and test set (n=49) using a single 80:20 split to retain most observations for model fitting while reserving a test set for exploratory assessment. The CEMP score could be calculated in 210 complete cases. Following training, the model was used to predict outcomes in the test set and subsequently calculate its accuracy. The 20% test set was reserved for exploratory performance assessment and was not used for hyperparameter tuning.

Model-attribution analysis: SHAP (SHapley Additive exPlanations) values were used to quantify the contribution of each feature and each patient-specific feature value to the XGBoost model output (Figure 4). SHAP is based on cooperative game theory and provides local and global model-attribution measures. SHAP analysis was implemented using the R package “SHAPforxgboost” (https://CRAN.R-project.org/package=SHAPforxgboost). SHAP values do not imply causal effects.

Construction of the C-19 vvECMO mortality prediction (CEMP) score: The SHAP value summary plots were analyzed in the first step. The position on the y-axis determining the feature’s importance was evaluated. For example, bilirubin had the highest mean absolute SHAP value (0.063). The mean absolute SHAP value analysis revealed that bilirubin (0.063), creatinine (0.052), BMI (0.028), and chronic lung disease (0.023) had the most impact on predicting outcome, while PEEP, lactate, dialysis pre-ECMO onset, and having a septic shock at ECMO initiation had mean absolute SHAP values rounded to 0.000 (Figure 4). Thus, features with the highest model attribution were considered as possible score candidates. In the next step, to determine how a low or high value of each parameter impacts the prediction outcome more or less, SHAP values of each patient per parameter were analyzed. Parameters are ordered on the x-axis by the SHAP value (each dot one value per patient), while the dot’s color represents the value of the feature from low (blue) to high (red). For example, a high value of bilirubin (red dots) had more impact on predicting mortality (SHAP value about 0.14) than lower bilirubin (blue dots) on predicting survival (SHAP value about -0.11). On the other hand, lower BMI values (blue dots) contributed to predictions of survival (-0.08) more than a high BMI value (red dots) contributed to predictions of mortality (0.03). Based on this information, a bilirubin value >3 mg/dl, a creatinine value >2 mg/dl, and chronic lung disease were identified as having the most impact on predicting mortality. At the same time, a BMI lower than 25 contributed to predictions of survival. Depending on the SHAP value on the x-axis, points ranging from -1 to 3 were attributed to the four identified parameters (Table 4), followed by risk class attribution. The thresholds and weights were derived from the observed SHAP distributions, were not prespecified, and may overfit the derivation cohort. Although the PaO₂/FiO₂ ratio and PaCO₂ ranked fifth and sixth, respectively, they were not included in the score. Mechanical ventilation parameters and arterial blood gas values had many missing values (Supplementary Tables 2 and 3). Finally, individual CEMP scores were calculated, and apparent performance was assessed (Figure 3 A, B; Table 2). The primary CEMP discrimination analysis was restricted to 210 complete cases; no multiple imputation or missing-data sensitivity analysis was performed.

**Exploratory multivariable assessment of CEMP components:**

Multivariable analysis was performed using the four CEMP score variables as input to estimate associations between the four CEMP components and mortality in the same complete-case cohort. Odds ratios, 95% confidence intervals, and p-values are listed in Table 2. All four components were associated with mortality in this cohort. Further, each of the remaining nine parameters initially identified as potential score candidates was tested in the multivariable analysis in addition to the four score parameters. Still, none proved to be independently associated with mortality.
